# Device infections related to cardiac resynchronization therapy in clinical practice–An analysis of its prevalence, risk factors and routine surveillance at a single center university hospital

**DOI:** 10.1002/clc.23620

**Published:** 2021-05-25

**Authors:** Bozena Ostrowska, Spyridon Gkiouzepas, Siri Kurland, Carina Blomström‐Lundqvist

**Affiliations:** ^1^ Department of Cardiology Uppsala University Uppsala Sweden; ^2^ Department of Medical Sciences and Cardiology Uppsala University Uppsala Sweden; ^3^ Department of Internal Medicine Uppsala University Uppsala Sweden; ^4^ Department of Infectious Diseases Uppsala University Uppsala Sweden

**Keywords:** cardiac resynchronization, complications, device, endocarditis, infection, pacemaker registry, pocket

## Abstract

**Background:**

The implantation rates of cardiac implantable electronic devices have steadily increased, accompanied by a steeper rise of device related infections (DRI).

**Hypothesis:**

The prevalence of DRI for cardiac resynchronization therapy (CRT) is higher in clinical practice than reported previously, even at a university hospital, and likely higher than reported to the national device registry.

**Methods:**

Electronic medical records of consecutive patients undergoing a CRT procedure between January 2016 and December 2017 were analyzed. Clinical history, procedure related variables and complications were reviewed by specialists in cardiology and infectious diseases.

**Results:**

A total of 171 patients, mean aged 74 years, 138 males (80.7%) were included. Twelve DRI occurred in 10 patients during mean 2.5 years follow‐up, giving a prevalence of 7% (incidence of 29/1000 person‐years). Reoperation, pocket haematoma, ≥3 procedures, previous device infection and indwelling central venous line were the strongest predictive factors according to univariate analysis. Out of 63/171 (36.8%) major complications, 31(49.2%) were lead‐related. There were 49/171 (28.7%) reoperations and 15/171 (8.8%) minor complications. The number major complications and DRI reported to the national device registry were 7/171 (4.1%) and 2/171 (0.6%), respectively, reflecting a 5‐fold underreporting.

**Conclusions:**

The high rate of CRT device infections is in sharp contrast to those reported by others and to the national device registry. Although a center specific explanation cannot be excluded, the high rates highlight a major issue with registries, reinforcing the need for better surveillance and automatic reporting of device related complications.

## 1 INTRODUCTION

The implantation rates of cardiac implantable electronic devices (CIED) have steadily increased along with their expanding indications^1^ and have been accompanied by an even steeper rise of device related infections (DRI).^2^ Although the reason for the increased infection rates remains unclear it may be related to the aging population with more comorbidities and fragility, and more complex devices, particularly for cardiac resynchronization therapy (CRT).^3^, ^4^, ^5^ The prevalence of infections is related to the complexity of the device and ranges between 1.6%/2 years follow‐up and 1.9%/3.4 years follow‐up for CRT‐P (P = pacemaker) and 3.1%/3.4 years follow‐up and 8.6%/2.6 years follow‐up for CRT‐D alone (D = defibrillator).^3^, ^6^ DRI are devastating complications resulting in increased morbidity, mortality and high costs for the national health care systems.^7^

Most of the reported DRI rates from registries and randomized clinical trials (RCTs) have ranged between 0.6% and 1.3% while those from prospective studies have reported somewhat higher rates ranging between 2.3% and 3.4%.^7^, ^8^, ^9^ The real‐world prevalence of DRI, as reflected by figures from device registries, has probably been underestimated due to a significant underreporting.^10^, ^11^ A meta‐analysis comparing complication rates for implantable cardiac defibrillator (ICD) procedures in RCTs versus the US National Cardiovascular Data Registry found an almost 3‐fold lower complication rate in registry data.^11^

The National Swedish Pacemaker and ICD Registry (www.pacemakerregistret.se) collect all CIED implantation procedures and related complications occurring during the first year after implantation from all 43 implanting sites in Sweden. According to the annually published report, the prevalence of CRT infections in Sweden and at our clinic has been 1.1%–1.2% and 0.6%–0.7%, respectively. Given the observed underreporting in other registries from previous publications^8^ our aim was to perform a retrospective, single‐center study of CRT‐device related infections at our university center and identify the most important predictive factors.

## 2 METHODS

### 2.1 Study population

All consecutive patients who underwent a CRT procedure (de novo, upgrade, revision or generator change) at Uppsala university hospital, which is a tertiary‐care hospital between January 2016 and December 2017 were included retrospectively to ensure at least 150–200 patients. There were no exclusion criteria. The 10 digit identity number of each patient, the type of CRT procedure and the reported complications were obtained from The National Swedish Pacemaker and ICD Registry.

The study protocol was approved by the Swedish Ethical Review Authority.

### 2.2 Study design

Patient's medical records were then analyzed in detail and obtained from the regional electronic medical record system (Cosmic, Cambio®), unified for recording of healthcare provided by all caregivers to patients in the referral area of Uppsala university hospital. Medical information from other regions were obtained from the responsible cardiologist by standardized telephone interviews. A thorough review of all patients' medical records was performed by two independent physicians and started from the index procedure, defined as the earliest CRT‐procedure performed between January 2016 and December 2017, and continued until September 30 in 2019, thus giving a follow up of at least 21 months. The index procedures were divided into three types: de novo (the very first CRT implantation), generator exchange and upgrade/revision. Demographics, comorbidities, medications, variables known as risk factors for device infection, procedures related variables and complications during follow‐up were collected.

The following national ICD‐10 (International Classification of Diseases −10) codes retrieved from the registry of diagnoses were used to cross‐check with the collected diagnoses of infections: T82.7 (Infection and inflammatory reaction due to other cardiac and vascular devices, implants and grafts); Y83.1 (Surgical operation with implant of artificial internal device as the cause of abnormal reaction of the patient, or of later complication); i33.0 (Acute and subacute infective endocarditis); A40‐41 (Other bacterial diseases); R65.1 (Systemic inflammatory response syndrome and infection) and R57.2 (Septic shock). All identified infections were reviewed, confirmed and if necessary redefined as a pocket infection, endocarditis or systemic infection (sepsis) in accordance with recently published guidelines^7^ by specialists in cardiology, electrophysiology and infectious diseases. The following data were registered for patients with DRI: the culprit device procedure, date and result of transesophageal echocardiography, microbiology, choice and duration of antimicrobial treatment and timing of hardware removal if performed. Superficial wound infections were excluded from this analysis. A culprit procedure was the device procedure judged as the most likely source of infection, not synonymous with the index procedure.

The presence of a post‐procedure complication was analyzed and defined as major if entailing a significant risk leading to reoperation, blood transfusion, prolonged hospital stay >24 h, intervention or readmissions for management, or death, and as minor if associated with patient discomfort, spontaneously resolving or treated on an outpatient basis.

A reoperation was defined as any surgical procedure performed to manage a postoperative complication during the follow‐up period. The index procedures were thus not classified as reoperations. Chest tubes or pericardial drainages were not classified as reoperations.

### 2.3 Outcomes

The primary endpoint was occurrence of DRI during the follow‐up period (prevalence in percentage of patients and incidence per 1000 person‐years). Conventional risk factors for device infection were tested and grouped as patient‐, procedure‐ and device‐related risk factors (Table S6) in accordance with previous reports.^7^ Patients with and without DRI were compared with regard to risk factors in order to identify those of greatest importance. Secondary endpoints were occurrence of other complications of the device surgery during follow‐up. For comparisons of complications between patient groups, only those occurring prior to the infection were included in the DRI group. Deaths directly related to the infection or indirectly due to withdrawal of required CRT were defined as DRI‐related mortality.

The prevalence of infections and other complications were compared with those reported to the National Swedish Pacemaker and ICD Registry for the study population.

### 2.4 Statistical analysis

Appropriate parametric statistical tests were used for the analysis of data. All continuous data were examined for normal distribution by Kolmogorov–Smirnov test. Numerical values with no normal distribution were presented as median values with range. Categorical variables were expressed as frequencies. Comparison of the categorical variables was performed using chi square test. Continuous data with non‐parametric distribution were compared using Mann–Whitney test. A *p*‐value <.05 was considered statistically significant. All statistical analyses were performed using the IBM SPPS version V.25.

## 3 RESULTS

### 3.1 Study population

A total of 171 patients, median age 74 (range 15–95) years, were included, 21 of whom were referrals from regional hospitals due to temporary lack of CRT‐implanters or were concomitant to a cardiac surgery or ablation. The patient characteristics are shown in Table 1. All patients had symptomatic heart failure and fulfilled conventional criteria for CRT.

**TABLE 1:** Patient characteristics

Characteristiscs	Patients *N* = 171
Age, median (range), years	74 (15–95)
Male sex	138 (80.7)
BMI, median (range)^a^	26.6 (15.1‐45.8)
Steroid treatment	7 (4.1)
Antithrombotic agents	142 (83.0)
Oral anticoagulants^b^	96 (56.1)
Antiplatelets^b^	58 (33.9)
Heparin/ LMWH	1 (0.6)
Chronic kidney disease (eGFR≤30)^c^	25 (14.6)
Diabetes mellitus^b^	36 (21.1)
COPD	17 (9.9)
Malignancy^b^	15 (8.8)
Chronic skin disease	8 (4.7)

*Note*: Figures are numbers and percentages are within bracket unless otherwise stated.

Abbreviations: BMI, body mass index; COPD, chronic obstructive pulmonary disease; eGFR, estimated glomerular filtration rate; LMWH, low molecular weight heparin; N, number.

^a^ 11 missing data.

^b^ 1 missing data.

^c^ 2 missing data.

### 3.2 Pre, peri‐, and intraprocedural routines

All patients had normal white blood cell count and C‐reactive protein prior to surgery.

A whole‐body shower with Chlorhexidine soap/shampoo twice with 6 h interval was undertaken prior to surgery. Standard intravenous (iv) antibiotic prophylaxis was Cloxacillin 2 g or Clindamycin 600 mg (in case of allergy) administered 0.5–1 h prior to incision, reiterated at 2 and 8 h after the initial dose for de novo/upgrade/revision operations. Antiplatelet therapy except for acetylsalicylic acid was withdrawn 5 days before the implantation if not absolutely indicated. Treatment with vitamin K antagonists was continued uninterrupted with an international normalized ratio (INR) <2.5 on the day of surgery while DOACs were interrupted for at least 24 h before surgery. One patient with left ventricular assist device (LVAD) had ongoing heparin treatment.

The hair in the surgical field was cut with clippers. Residuals of monitoring electrodes were removed and one additional wash with Chlorhexidine soap was performed. The skin was then painted with a Chlorhexidine‐alcohol solution. Adhesive sheets were used to cover the patient. All operators had full aseptic body gowning and double gloving.

All procedures were performed under local anesthesia and conscious sedation, using axillary/subclavian vein access. A venography of the coronary sinus was obtained in all patients before implanting the left ventricular lead. Each device was secured with a suture and the wound was closed with multiple layer sutures.

### 3.3 Procedures

There were in total 220 procedures: 171 index procedures and 49 reoperations due to complications (including nine extractions/explantations). The majority of the 171 CRT index procedures were de novo implantations (70%) with an even distribution between CRT‐P and CRT‐D (Figure S1). The vast majority, 196/220 (89.1%) of the procedures, including 128/132 (97%) de novo/upgrade/revision index procedures, 24/39 (62%) generator replacements and 44/49 (90%) reoperations were performed by three cardiologists. The remaining 24/220 (10.9%) procedures including 4/132 (3%) de novo/upgrade/revision index procedures, 15/39 (38%) generator replacements and 5/49 (10%) reoperations were performed by six other implanters. All CRT systems were transvenous using an infraclavicular subcutaneous pocket.

A total of 207/220 (94%) of the procedures were performed in the EP laboratory during office hours and 13/220 (6%) in the operating room for thoracic surgery.

The mean index procedure duration was 99 min (42–410 min) for de novo implant, 87 min (25–138) for upgrade/revision and 27 min (15–73 min) for generator change.

### 3.4 CIED infections

A total of 12 DRI occurred in 171 patients during a mean of 29.6 ± 11 months follow‐up (414 person‐years). The 12 DRI (10 endocarditis/ sepsis and 2 pocket infections) occurred in 10 patients giving a prevalence of 7%, translating to an incidence of 29/1000 person‐year. Nine DRI occurred within 1 year, directly related to the culprit procedure.

Of the 12 DRI, six were classified as possible and six as definite, according to the EHRA criteria for device related infections.^7^ Nine of 12 blood cultures were positive and S aureus was the most common agent (42%) (Table 2). Of the three DRI with negative blood cultures, two had bacterial growth from the pocket alone and one with sepsis clinically was analyzed during ongoing treatment with antibiotics. Lead/valve vegetation was seen in 3 of 11 (27%) patients undergoing transesophageal echocardiography.

**TABLE 2:** Results of the microbiology tests in the 10 patients with device related infections

Blood culture	Pocket culture	Time to infection^a^ (months)	TEE	Type of device infection	Deaths related to infection	Device – lead extracted
Neg	*E. coli*	<1	0	Pocket	No	Yes
Neg^b^	S. Epidermis	<1	0	Pocket infection	No	Yes
Listeria^b^	No growth	18	0	CIED/IE possible	No	Yes
*E. faecalis*	No growth	17.5	Veg	CIED/IE definitive	Yes^c^	Yes
*S. aureus* + Beta G Streptococcus^d^	No growth	1	0	CIED/IE definitive	No	Yes
S. Aureus^d^	No growth	1	Veg	CIED/IE definitive	Yes^c^	Yes
S. Aureus	No growth	<1	0	CIED/IE definitive	No	Yes
S. Aureus	No growth	<3	0	CIED/IE possible	Yes	No
S. Aureus	No growth	5	NP	CIED/IE possible	Yes	No
Neg (during antibiotics)	No growth	<1	0	CIED/IE possible	Yes	No
Beta B Streptococcus	No growth	24	Veg	CIED/IE possible	No	Yes
S. Epidermis	No growth	1	0	CIED/IE possible	No	Yes

*Note*: 0: No vegetation or no microbiological growth.

Abbreviations: CIED, cardiac implantable electronic device; E, enterococcus; IE, infectiv endocarditis; NP, not performed; S, staphylococcus; TEE, transesophageal echocardiography; Veg, vegetation.

^a^ From culprit procedure to diagnosis of infection.

^b^ Same patient.

^c^ Due to withdrawal of required CRT after device extraction/explantation.

^d^ Same patient.

All DRI patients were treated with antibiotics according to ESC guidelines.^7^ Hardware removal was performed in 9 of 12 (75%) DRI while salvage antibiotic therapy was used alone in the remaining three due to severe comorbidities.

### 3.5 Risk factors for device infection

The predefined risk factors in infected and non‐infected patients are shown in Tables 3 and 4.

**TABLE 3:** Comparison of risk factors for device related infections in patients with and without device related infections

Risk factors for device related infection	Non‐DRI patients *N* = 161	DRI patients^a^ *N* = 10	*p*‐value
Patient‐related			
Age, median (range)	73 (15–95)	65 (52–85)	.03
Sex, male	128 (79.5)	10 (100)	.11
BMI, mean (range)	26,7 (15.1–45.8)	26 (20,8‐35,3)	.60
Diabetes mellitus	34 (21.3)	2 (20)	.96
Chronic kidney disease^b^	23 (14.5)	2 (20)	.63
COPD	17 (10.6)	0 (0)	.28
Malignancy	12 (7.5)	2 (20)	.16
Chronic skin disease	8 (5)	0 (0)	.47
Antithrombotic medication (ongoing):	133 (82.6)	9 (90,0)	.55
Antiplatelet agents	54 (33.8)	4 (40)	.69
Oral anticoagulants	91 (56.9)	5 (50)	.67
Heparin/LMWH	0 (0)	1 (10)	<.001
Corticosteroid treatment	5 (3.1)	2 (20)	.009
Fever<24 h before surgery	0 (0.0)	0 (0,0)	NA
Previous CIED infections	0 (0)	2 (20)	<.001
Temporary pacemaker/central venous line	3 (1.9)	4 (40)	<.001
Left ventricular assist device	0 (0)	2 (20)	<.001
Procedure‐related			
>3 personnel during surgery	115 (73.2)	7 (70)	.82
Procedure time (min), median (range)^c^	80 (15–410)	97 (36–158)	.25
Procedure type^d^			
De novo	101 (62.7)	6 (60)	.87
Upgrade/revision	22 (13.6)	3 (30)	.29
Generator change	38 (23.6)	1 (10)	.20
≥3 prior procedures	4 (2.5)	4 (40)	<.001
Reoperations	24 (14.9)	4 (40)	.037
Lead‐related complications	18 (11.2)	4 (40)	.008
Cardiac perforation	2 (1.2)	1 (10)	.038
Pocket haematoma^e^	1 (0.6)	2 (20)	<.001
Device‐related			
CRT‐D	86 (53.4)	10 (100)	.0033

*Note*: Figures are numbers of patients with percentage in brackets unless otherwise stated.

Abbreviations: BMI, body mass index; CIED, cardiac implantable electronic devices; COPD, chronic obstructive pulmonary disease; CRT‐D, cardiac resynchronization therapy‐defibrillator; DRI, device related infections; LMWH, low molecular weight heparin; Min, minutes; NA, not applicable.

^a^ All complications/reoperations listed are those occurring/performed prior to DRI. Remaining major complications are presented in Table 4.

^b^ Defined as eGFR≤30.

^c^ Time from the start of the skin incision until the wound closure.

^d^ The procedure type refers to the culprit procedure in the DRI‐group while it represents the last type of procedure in an individual patient at the end of follow‐up in the non‐DRI group. This means that “de novo” here are de novo index procedures with no reoperation during follow‐up.

^e^ Requiring reoperation or blood transfusion.

**TABLE 4:** Complications of CRT procedures and mortality in patients with and without device related infections

	Number of complications per patient group		
Type of complication	Non‐DRI group (161 patients)	DRI group^a^ (10 patients)	*p*‐value
Any complication	52 (32.4)	6 (60)	.0726
Reoperation^b^	30 (18.6)	7 (70)	.0001
Patients with >1 complication	36 (22.4)	4 (40)	.2023
Major complications:	37 (23)	6 (60)	.0088
Lead‐related:	27 (16.8)	3 (30)	.2872
Failed lead implantation	10 (6.2)	0 (0)	.4171
Lead dislogment	17 (10.6)	3 (30)	.0739
Perforation	2 (1.2)	1 (10)	.0375
Without tamponade	1 (0.6)	0 (0)	.8028
With tamponade	1 (0.6)	1 (10)	.0074
Pocket revision	3 (1.8)	0 (0)	.6635
Pocket heamatoma	1 (0.6)	2 (20)	.0001
Deep vein thrombosis	2 (1.2)	0 (0)	.7232
Pneumothorax requiring drainage	1 (0,6)	0 (0)	.8028
Stroke	0 (0)	0 (10)	NA
CS dissection	1 (0.6)	0 (0)	.8028
Minor complications:	15 (9.3)	0 (0)	.3127
Wound infection treated with antibiotics	3 (1,8)	0 (0)	NA
Pocket Heamatoma^c^	7 (4.2)	0 (0)	NA
CS dissection^d^	5 (3.0)	0 (0)	NA
Mortality:			
Deaths ‐ complication or DRI‐related:	0 (0)	5 (50)	NA
Within 3 months	0 (0)	2 (20)	NA
Beyond 3 months	0 (0)	3 (30)	NA

*Note*: Figures represent number of complications with percentages of number of patients in brackets unless otherwise stated.

Abbreviations: CRT, cardiac resynchronization therapy; CS, coronary sinus; DRI, device related infection; N, number of complications; NA, not applicable.

^a^ The complications in the DRI group are those that occurred prior to the DRI;

^b^ Reoperations were per definition due to complications.

^c^ Not requiring intervention or blood transfusion;

^d^ Without pericardial effusion and not impeding the procedure.

Previous DRI, an indwelling central venous line, periprocedural heparin, reoperations, ≥3 procedures and pocket haematoma were the strongest risk factors for DRI (*p* ≤ .001) in the present study. There were four patients with major haematomas of which 1/4 (25%) was treated with warfarin, 2/4 (50%) with platelet inhibitors and 1/4 (25%) was on heparin. The rate of DRI among the three main operators were 8/72 patients (11.1%) for the first operator, 4/42 patients (9.5%) for the second operator and 0/38 patients (0%) for the third operator. No DRI occurred for the other six additional operators performing 24/220 (10.9%) operations.

Major complications occurred in 37/161 (23%) of the patients in the non‐DRI group and in 6/10 (60%) of patients in the DRI group. A comparison between patients with and without DRI with regard to surgical complications is presented in Table 4.

#### 3.5.1 Overall surgical complications during follow‐up

A total of 78/171 (45.6%) complications (including DRI), 63 (80.8%) of which were major, occurred in 171 patients. Out of 63 major complication, 31 (49.2%) were lead‐related. Forty (23.4%) of the patients experienced at least one major or minor complication. There were 49/171 (28.7%) reoperations, performed in 29 patients.

Among the de novo/upgrade/revisions, that is, complex procedures including lead manipulations, performed in 132 patients, there were 60/132 (45.5%) major complications, of which 31/60 (51.7%) were lead‐related. A total of 48/132 (36.4%) reoperations were performed in this group. In the generator exchange group of 39 patients, there were 3 /39 (7.7%) major complications and 1/39 (2.6%) reoperation.

### 3.6 Mortality during follow‐up

A total of 34 patients (19.9%) died. Fifteen (44.1%) of these deaths were related to CRT. Five of the latter deaths occurred among the 10 (50%) DRI‐patients, who died of endocarditis/sepsis, 3 of whom received salvage therapy alone. The other 10/15 deaths occurred in the non‐DRI patients due to deterioration of heart failure after the CRT implantation.

### 3.7 Comparison with national device registry data

A total of 9/171 (5.3%) DRI occurred within 1 year from the culprit procedure but only 2/171 (1.2%) were reported to the national device registry, reflecting a 4.5‐fold underreporting. No procedure or complication related deaths were reported to the national registry, although five DRI‐related deaths occurred. There were 59/171 (34.5%) overall major complications during 1 year from the procedure although only 9/171 (5.3%) were reported to the national registry (Table 5).

**TABLE 5:** Total number of complications of CRT procedures during follow‐up and reports to the national device registry

Type of complication	Overall complications (in 171 patients)	Reported to registry (in 171 patients)
Major complications	63 (36.8)	9 (5.2)
Lead‐related	31 (18.1)	6 (3.5)
Pocket revision or heamatoma	7 (4.1)	1 (0.6)
Myocardial perforation, CS dissection or pneumothorax requiring drainage	5 (2.9)	0 (0.0)
Stroke or deep vein thrombosis	3 (1.8)	0 (0.0)
Device related infection	12 (7.0)	2 (1.2)
Deaths ‐ complication‐ or DRI related	5 (2.9)	0 (0.0)
Minor complications^a^	15 (8.8)	0 (0.0)

*Note*: Figures represent number of complications with percentage of the number of patients in brackets unless otherwise stated.

Abbreviations: CRT, cardiac resynchronization therapy; CS, coronary sinus; DRI, device related infection; N, number of complications; NA, not applicable.

^a^ See Table 4 for details.

## 4 DISCUSSION

The observed prevalence of 7% DRI in CRT recipients (incidence of 29/1000 person‐years) after mean 2.5 years follow‐up is higher than previously reported.^3^, ^6^, ^7^, ^9^ Lower rates (1%–3%) have been reported from registries^8^ and randomized trials^12^, ^13^ while higher (4.2%–4.8%) from observational studies.^6^ To the best of our knowledge, in the few studies of CRT procedures using data from case records^6^, ^14^ a 5% CIED infection rate (15/1000 person‐years) was regarded as high.^14^ The prevalence of device infections increases with the complexity of the device, as illustrated by the lower rates of DRI for CRT‐P, ranging between 1.6%–1.9%/2–3.4 years follow‐up^16^ and the higher rates for CRT‐D alone from 3.1%/3.4 years to as high as 8.6%/2.6 years follow‐up.^3^, ^6^ In a recent Danish device registry study, the DRI incidence was 2.18% for CRT‐P and 3.35% for CRT‐D during a device lifetime, whereas it was 4.38/1000 and 6.76/1000 device‐years after de novo CRT‐P and CRT‐D, respectively, but higher for replacements, 11.56/1000 and 18.94/1000 device‐years, respectively.^5^ The figures may represent low estimates since DRI were only counted in patients with their system removed due to infection. Although registries are prone to underreporting of complications as well as misclassifications, the Danish registry is regularly audited and considered to have a high validity.

Although one cannot exclude a center‐ or surgical technique specific explanation for the high prevalence of DRI in the present study, one likely explanation is the meticulous data review of electronic charts capturing all DRI and thus overcoming cases with incorrect or missing ICD‐10 codes. As compared to other studies, patient demography seemed similar, which argues against a selection of patients with more severe comorbidities or anatomical/surgical difficulties. Still, the present study confirmed several previously described modifiable risk factors, the strongest being reoperations, pocket haematoma, cardiac perforation and indwelling central venous line.

Reoperation, particularly multiple reoperations, is a well‐known strong risk factor for DRI.^7^, ^9^ Pockets can be colonized by bacteria even after minor reoperations. The total rate of observed reoperations (28.7%) after all CRT procedures and the rate after de novo/upgrade/revision procedures (36.4%) was unexpectedly high in the present study as compared to prior reports with the highest figure of 9%.^15^ The observed rate of major complications (36.8%) was higher than reported previously for CRT‐procedures, in the Danish registry 6.7%–11%, and was strongly associated with DRI.^8^ The vast majority of major complications were lead revisions mainly due to lead dislodgements, which is a well‐known and strong risk factor for DRI.^9^ The 18.1% observed frequency of lead‐related complications in the present study is high in comparison with previous reports, including a Danish registry in which the risk for any lead complication was 3.6%.^15^

A Danish registry study further demonstrated that the operator inexperience was an independent risk factor with an adjusted risk ratio of 1.9 (95% confidence interval 1.4–2.6) for any complication if performed by an operator with an annual volume < 50 procedures.^8^ In the present study, the three main operators met the criteria for being experienced based on their annual volume of procedures. The possibly higher rate of DRI for 2 of the operators may be related to differences in procedure time, uneven load of case complexity, variable skills or to nosocomial infection, the latter not analyzed. A low annual hospital device implant volume was also reported as a risk factor for DRI by the Danish registry study with higher complication rates in centers with less than 750 implantations annually.^8^ Given the high volume of annual CIED procedures in our hospital, this can unlikely explain the high rate of DRI. Out‐of‐hours procedures increases the risk by 1.5,^8^ but was not a risk factor in the present study.

Post‐procedural pocket haematoma, known to increase the risk of infection by nine‐fold,^16^ was a strong risk factor in our study as well. Pocket haematoma can be prevented by avoiding bridging heparin/low molecular weight heparin therapy, known to increases the risk for haematoma by 5‐fold,^17^ minimizing broad incisions and withdrawing dual platelet inhibitors if possible a week before surgery.^7^ Although oral anticoagulation and antiplatelets were widely used in the present study, bridging was never adapted and heparin was limited to one patient with LVAD. Antibacterial envelopes can prevent pocket infection,^13^ but were not routinely used in the present study.

Procedure duration, known to be correlated with DRI,^7^, ^9^ has been shown to increase the risk of infection stepwise as compared to durations <30 min, and increases 1.5 times for durations 60–90 min and 2.4 times for those exceeding 120 min.^5^ Procedure duration was not a risk factor in the present study possibly due to the wide variation of procedure times even for the same procedure types. Longer procedure time are usually a reflection of more difficult cases, but whether such observation is related to the operator skill or complexity of device/patient characteristics was difficult to evaluate in the present report. The complexity of CRT procedures at our center was likely comparable to other university centers as we use the same indications for implant. The small study population precluded us from performing more complex statistics of the correlation between the procedure times and the rate of complications.

A well‐recognized and modifiable risk factor for DRI is indwelling central venous line,^7^ which was present in 4.1% of our patients and in nearly half of the DRI cases. Removal of all central venous lines should always be considered before device surgery. Steroid treatment, observed in 4.1% of the patients in the present study, is also a known risk factor for DRI but difficult to withdraw and usually implies another coexisting disease.

CRT‐D, being a larger and bulkier device, has been associated with a double risk^3^ or even nearly 5.5‐fold higher risk for DRI as compared with CRT‐P.^9^ The present study confirmed these findings as all patients with DRI had CRT‐D implanted.

The high rate of major complications (45.5%) in the de novo/upgrade/revision group exceeded by far figures of 11% and 17% reported by others for CRT‐P and CRT‐D, respectively.^18^, ^19^ While it is crucial to reconsider an indication for the reoperation, it remains unproven if such precautions could have reduced the infection rate in the present study.

The 2.9% device related mortality in the present study, being entirely due to DRI, was higher than in a previous report of one procedure‐related death in 5942 patients^8^ and 0.8% in another study.^3^ The inconsistencies in reports and definitions of mortality rates after CIED procedures makes comparisons difficult. DRI are associated with high fatality rates ranging from 6%–15% at 1 year to 14%–33% at 3 years,^4^ even after device system extractions.^7^, ^20^ In the present study, the 50% mortality for DRI, mainly related to the salvage therapy, is high compared to previous reports^7^, ^20^ with only one study reporting similar rates.^14^

Even though the small size of the cohort is a limitation and a center specific explanation for the high rates of infection cannot be excluded, they could be explained by the extensive chart review performed by specialists in electrophysiology and infectious diseases. The present findings of underreporting to the national device registry are in line with many others,^10^, ^11^ and reflect the problems with registries being voluntary, lacking monitoring routines and procedures for continuous feedback. At the hospital level, regular and shared responsibilities for internal quality controls may further increase awareness and reduce a reluctance to report complications.

The findings in the present study should not be interpreted as a questioning of the benefits of CRT. On the contrary, the results emphasize the importance of both recognizing the risks of DRI and of allocating resources towards their prevention.

## 5 CONCLUSIONS

The high prevalence of device infections is in sharp contrast to those reported previously, and to those reported to the national device registry. Although a center specific explanation cannot be excluded, the high rates could also be due to the extensive chart review and thereby highlight a major issue with present registries, reinforcing a need for automatic and mandatory systems for reporting of device related complications. Hopefully it will give others an incentive to perform similar surveillances.

## 6 STUDY LIMITATIONS

The small cohort of CRT implantations collected retrospectively, may have missed minor complications not recorded in the medical records and precluded a multivariate analysis.

## CONFLICT OF INTEREST

Bozena Ostrowska, Spyridon Gkiouzepas, and Siri Kurland have nothing to disclose. Carina Blomstrom‐Lundqvist reports personal fees from Bayer, Medtronic, CathPrint, Octopus, Sanofi Aventis, Boston Sci, and Merck Sharp and Dohme outside the submitted work.

## Supporting information


**Supplementary figure 1** Types of index procedures at inclusion
CRT‐P=Cardiac Resynchronization Therapy Pacemaker; CRT‐D=Cardiac Resynchronization Therapy Defibrillator

**Supplementary table 6** Analyzed and predefined risk factors for device related infections
*defined as estimated glomerular filtration rate (eGFR) ≤ 30;
**pacemaker(‐P) or defibrillator(‐D)
BMI = Body Mass Index; CRT = Cardiac Resynchronization Therapy; h = hours; LMWH = Low Molecular Weight Heparin; PM = Pacemaker

## Data Availability

The data underlying this article cannot be shared publicly unless a new application to the ethical committee is made and permission obtained. The data can be shared on reasonable request to the corresponding author.
