# Supplementary figures and images for: Device infections related to cardiac resynchronization therapy in clinical practice–An analysis of its prevalence, risk factors and routine surveillance at a single center university hospital
Source: Clin Cardiol. 2021 May 25;44(6):739–47. doi: 10.1002/clc.23620 (PMC8207984; doi:10.1002/clc.23620)

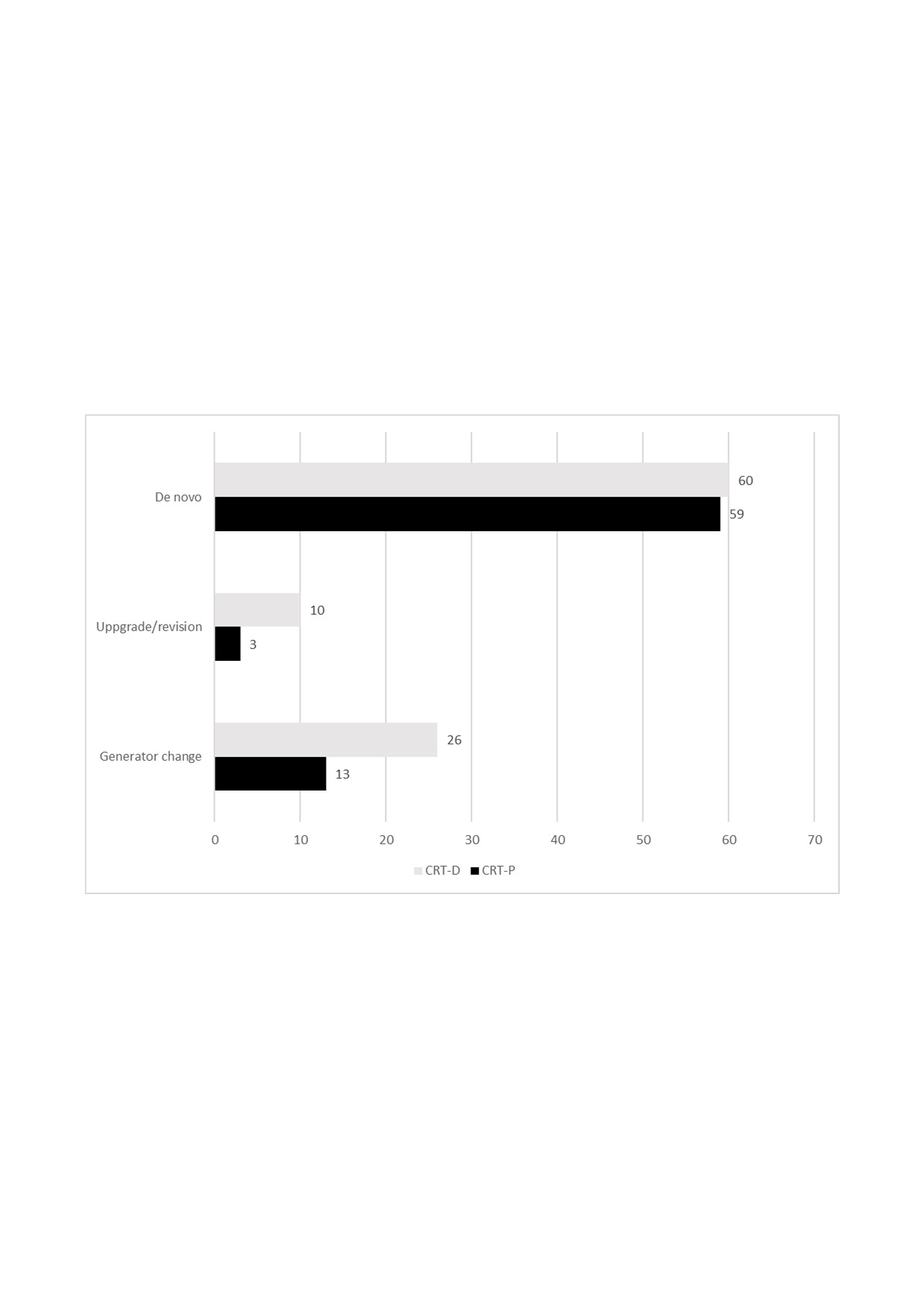

Supplement: Supplementary file 1 — Supplementary figure 1 Types of index procedures at inclusion CRT‐P=Cardiac Resynchronization Therapy Pacemaker; CRT‐D=Cardiac Resynchronization Therapy Defibrillator [file CLC-44-739-s001.jpg]
