# Supplementary material for: Device infections related to cardiac resynchronization therapy in clinical practice–An analysis of its prevalence, risk factors and routine surveillance at a single center university hospital
Source: Clin Cardiol. 2021 May 25;44(6):739–47. doi: 10.1002/clc.23620 (PMC8207984; doi:10.1002/clc.23620)
Supplement: Supplementary file 2 — Supplementary table 6 Analyzed and predefined risk factors for device related infections *defined as estimated glomerular filtration rate (eGFR) ≤ 30; **pacemaker(‐P) or defibrillator(‐D) BMI = Body Mass Index; CRT = Cardiac Resynchronization Therapy; h = hours; LMWH = Low Molecular Weight Heparin; PM = Pacemaker [file CLC-44-739-s002.docx]

| Patient-related | Procedure-related  prior to DRI | Device-related |
| --- | --- | --- |
| Age | Procedure time | CRT-type** |
| Sex | >3 personnel during the procedure |  |
| BMI | Reoperations |  |
| Diabetes mellitus | 3 or more procedures |  |
| Chronic Kidney Disease* | Lead revisions |  |
| Malignancy | Major complications |  |
| Chronic skin disease | Pocket haematoma |  |
| Fever < 24 h before surgery | Lead perforation |  |
| Ongoing anticoagulants/  antiplatelets | Type of culprit procedure:   - De novo - Generator exchange - Upgrade/revision |  |
| Corticosteroid treatment | Operator |  |
| Previous infection during follow-up |  |  |
| Temporary PM/indwelling central venous line |  |  |
| Left ventricular assist device |  |  |
|  |  |  |
